# SARS-CoV-2 Infection—A Trigger Factor for Telogen Effluvium: Review of the Literature with a Case-Based Guidance for Clinical Evaluation

**DOI:** 10.3390/life13071576

**Published:** 2023-07-18

**Authors:** Gabriela Mariana Iancu, Estera Molnar, Loredana Ungureanu, Simona Corina Șenilă, Adrian Hașegan, Maria Rotaru

**Affiliations:** 1 Department of Dermatology, Faculty of Medicine, Lucian Blaga University of Sibiu, 550169 Sibiu, Romania; 2 Clinic of Dermatology, County Emergency Hospital of Sibiu, 550245 Sibiu, Romania; 3 Department of Dermatology, “Iuliu Hațieganu” University of Medicine and Pharmacy, 400006 Cluj-Napoca, Romania; 4 Department of Dermatology, Emergency County Hospital, 400006 Cluj-Napoca, Romania; 5 Department of Urology, Faculty of Medicine, Lucian Blaga University of Sibiu, 550169 Sibiu, Romania; 6 Clinic of Urology, County Emergency Hospital of Sibiu, 550245 Sibiu, Romania

**Keywords:** telogen effluvium, COVID-19, diffuse reversible alopecia

## Abstract

Telogen effluvium post-COVID-19 is a condition characterized by the diffuse and reversible loss of scalp hair in the period following infection with SARS-CoV-2, and it is currently the second cause of alopecia in women. In the context of the COVID-19 infection, intense psychological stress contributes to alopecia appearance, along with systemic inflammation, autoimmune reactions, oxidative stress, and virally induced hypoxia. Cytokines with proinflammatory action and vasoactive substances negatively modulate the metabolism of some molecules, such as proteoglycans, involved in the hair follicle’s growth cycle. Studies show that a large percentage of hairs will suddenly enter the catagen phase during a moderate to severe COVID-19 infection. In the present paper, we update the data from the literature with a clinical example. Our case highlighted that the telogen effluvium after infections with SARS-CoV-2 is reversible with appropriate dermatological treatment. For therapeutic success, informing the patient about this pathology’s self-limited and reversible character is essential to reduce the emotional stress that may aggravate the disease.

## 1. Introduction

Infection with SARS-CoV-2, which causes severe acute respiratory syndrome and post-COVID-19 syndrome (which includes all complications occurring at least four weeks after the SARS-CoV-2 infection has been cured), has a major impact on all medical specialties [1,2,3]. In Europe, the first increased incidents of telogen effluvium were reported during the Italian quarantine in March 2020. In most patients, an increase in preexisting hair loss was observed, and a decisive role in this regard was played by mental stress (imposed by the quarantine period and the uncertainty of the disease’s evolution) but also by therapy with recombinant IFN-α 2β in those who required care measures in intensive care units [4].

Initially, the described skin manifestations of COVID-19 infections were few; later, they were analyzed and classified. These can be virus-related and virus-treatment-related [5]. The most frequent dermatological manifestations described in positive COVID-19 patients were:

- maculopapular lesions,
- urticaria-like rash (average duration of four days),
- morbilliform rash (average duration of seven days),
- papulosquamous eruption (average duration of 20 days),
- pityriasis rosea Gibert,
- erythema multiforme-like rash,
- varicella-like rash,
- transient livedo reticularis,
- acro-ischemic lesions (pseudo chilblain or COVID-19 fingers) with a clinical appearance of acral pernio-like or acral ischemia with a vascular–occlusive appearance (due to the transient increase of antiphospholipid antibodies or by activating the prothrombotic status) [5,6,7,8,9,10,11,12,13].

In addition, virus-treatment-related dermatoses have also been described (due to the antiviral, antibiotic, and antimalarial therapies used):

- urticaria,
- angioedema,
- erythroderma,
- generalized pustular reactions after the administration of Hydroxychloroquine,
- drug toxidermia,
- Stevens–Johnson syndrome or toxic epidermal necrolysis syndrome [5].

Some skin manifestations are more frequent in severe forms of the disease. Still, a direct causal relationship between the severity of the viral infection and the skin manifestations has not been established [5,14,15].

Over time, it was observed that the latest variants of the SARS-CoV-2, delta, and omicron have a less aggressive profile, including at the skin level (17.6% of patients with the delta version developed skin manifestations and 11.4% developed skin manifestations for the omicron version, versus 20.4% for the alpha version, as reported by Recalcati et al. in 2020) [16,17].

The stress induced by the SARS-CoV-2 infection is involved in triggering acute type of dermatoses (herpes simplex, herpes zoster, alopecia areata, etc.) or the exacerbation of pre-existing dermatoses (psoriasis, seborrheic dermatitis, allergic dermatitis, etc.) [14].

During the convalescence and post-COVID-19 period, there have been described cases of livedo reticularis (1 month after viral infection explained by viral coagulopathy), psoriasis exacerbation, telogen effluvium (TE), seborrheic dermatitis, pseudo chilblains lesions on the limbs (as a manifestation of the delayed immune reaction in those genetically predisposed, appearing after approximately three weeks), allergic, or irritant contact dermatitis [14,18].

Post-COVID-19 telogen effluvium is a frequent, self-limiting dermatological pathology, which is completely reversible 6–12 months post-infection [19]. However, it has major aesthetic, psychological, and social implications among the general population, especially women. Hussein et al. observed that diffuse hair loss occurred approximately two months after the infectious episode with COVID-19, especially in moderate and severe forms of the disease, compared to the classic acute form of telogen effluvium, which occurred after events that induced intense stress, postpartum, or post-medication [19]. Monari et al. highlighted, in a study on 96 patients infected with SARS-CoV-2, that there was no association between TE and the days of fever, hospitalization, and COVID-19 positivity [20].

The pathogenic mechanisms involved in the appearance of post-COVID-19 telogen effluvium include both the action of pro-inflammatory cytokines in the context of severe inflammation, as well as the viral coagulopathy that causes the formation of microthrombi at the level of the capillary microcirculation of the hair follicle, but also the direct cytopathic effect of the virus on the hair follicles [4,21,22].

To establish the diagnosis, it is essential to perform a detailed anamnesis that highlights the excessive and diffuse hair loss in the post-infection period with SARS-CoV-2, which must be corroborated with the dermatological and trichoscopic examination. Trichoscopy reveals the diffuse reduction of hair density, short hairs, and multiple bare follicles in places, which present aspects similar to the classic forms of TE [19].

The long-term prognosis is similar to classic telogen effluvium, with the restoration of capillary density after removing the causative agent, and a major role is played by the patient’s psychological support.

We present the case of a patient diagnosed and treated for post-COVID-19 telogen effluvium in our hospital in 2022.

## 2. A Case-Based Guidance for Clinical Evaluation

A 48-year-old non-smoker female patient with arterial hypertension and chronic venous disease presented with excessive and diffuse hair loss from the scalp in a short time and with changes in hair texture. The onset was two months later after a moderate form of SARS-CoV-2 infection.

Anamnesis excluded other possible etiologies of TE, such as:

- restrictive diets,
- medications (for ten years, she had been treated with venotonics and converting enzyme inhibitors; for COVID-19 infection, she only took antipyretic drugs),
- chemical substances that can be involved in shortening the duration of the anagen phase,
- other stressful events outside of the COVID-19 infection (surgical interventions, mental, or physical stress, etc.),
- menstrual cycle disorders (to confirm or exclude other endocrinological causes that could be the basis of excessive hair loss).

**Figure 1:**
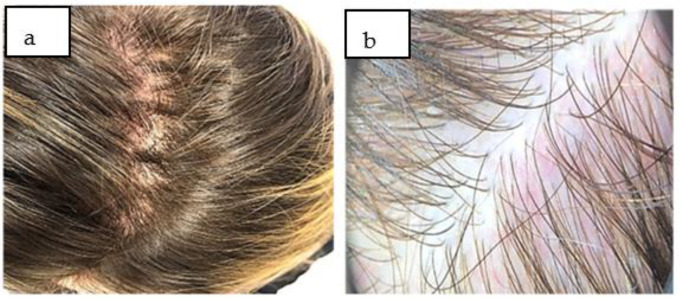
(**a**) Diffuse hair lost in frontoparietal areas with a non-scarring pattern at first examination. (**b**) Diffuse decrease of hair density, with thin hairs and without scales in the examined area (trichoscopic aspect).

Clinically, at the time of the examination, a diffuse thinning of the hairs on the scalp was evident and more accentuated in the frontotemporal area, with the scalp having a normal appearance (Figure 1a). Affirmatively, at home, the patient applied various anti-hair loss lotions and shampoos without improvement.

To support the diagnosis of the post-COVID-19 telogen effluvium, the following were important:

- Anamnesis: In the last two months, she had a moderate form of SARS-CoV-2 infection;
- The dermatological examination: This was a diffusely low density of hairs, normal scalp skin, and no subjective symptomatology. The hair pull test was positive;
- Trichoscopy: There was a diffuse decrease in hair density; in some places were bare follicles at the level of the affected areas, without scales on the examined fields (Figure 1b), which confirmed the diagnosis of telogen effluvium and excluded other conditions that evolve with non-scarring alopecia.

To be able to establish the diagnosis of telogen effluvium after COVID-19, we needed to exclude the most important types of non-scarring alopecia:

- Alopecia areata, in which circumscribed, non-scarring, noninflammatory, asymptomatic alopecia plaques appear. Trichoscopically noted features are present exclamation mark hairs, yellow or black dots, and smaller vellus hair. Spontaneous recovery is obtained in most cases after the elimination of the causative agent [23,24].
- Androgenetic alopecia in women with a diffuse decrease in hair volume, wherein the frontoparietal area of the scalp is the most affected, but with the frontal line remains intact. Associated clinical signs of hyperandrogenism may be present (hirsutism and menstrual cycle disorders). Trichoscopy shows follicular miniaturization, the presence of yellow dots, and perifollicular pigmentation, and the ratio of telogen/anagen strands is <3:1 [25,26].
- Secondary syphilis, in which the alopecia may have a moth-eaten (parieto-occipital region) or a diffuse or mixed appearance. Trichoscopy is non-specific and may reveal empty hair follicles and smaller hairs follicles (TE-like), broken hairs, black dots (tinea capitis-like), zigzag hairs (alopecia areata-like), etc. The skin of the scalp is unaffected, and the serology for treponema pallidum is positive [27,28].
- Noninflammatory tinea capitis, in which the alopecia plaques extend to the periphery, are itchy, have scales on the surface, and the parasitic hairs break at the level of the scalp at the opening of the follicles with the appearance of black dots (tinea capitis caused by trichophyton) or at 1–3 mm above the scalp (tinea capitis caused by microsporum). Trichoscopy reveals black dots and/or comma hair and/or short, broken hair with perifollicular scaling. The mycological examination confirms the diagnosis, thus specifying the mycotic etiology [29,30].
- Drug-induced alopecia, which highlights a causal association or relationship between the administration and a new drug [31].
- Trichotillomania is an obsessive-compulsive psychiatric disorder characterized by repeatedly pulling out one’s hair from any region of the body. The pull-test is negative and tricoschopically may present a V-sign, trichoptilosis, hook hairs, broken hair, flame hairs, coiled hair, tulip hairs, hair powder, follicular micro-hemorrhages, etc. [32,33].

Our patient was treated with vitamin supplements (three months), topical Minoxidil 2% solution twice per day (six months), and hair-fortifying shampoos used twice a week for the long term. The patient received psychological support by confirming that this was a self-limiting condition, with the possibility of complete recovery of the capillary density.

**Figure 2:**
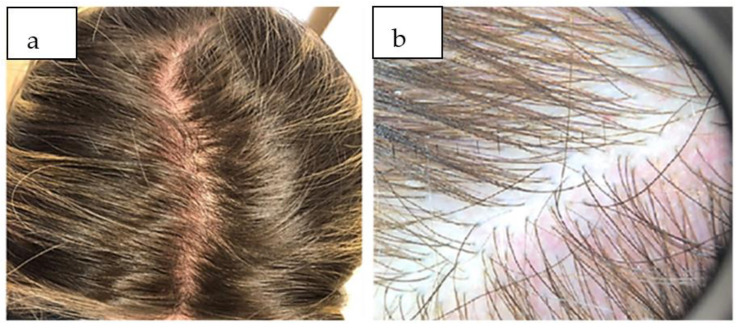
(**a**) After three months of treatment, showing partial recovery of TE. (**b**) Trichoscopic aspect three months after treatment, with a slow recovery of hair density.

The clinical and trichoscopic evaluation at three months follow-up highlighted the partial recovery of the capillary density, which required the maintenance of topical therapy with minoxidil and fortifying shampoo for another three months to consolidate the results (Figure 2a,b).

**Figure 3:**
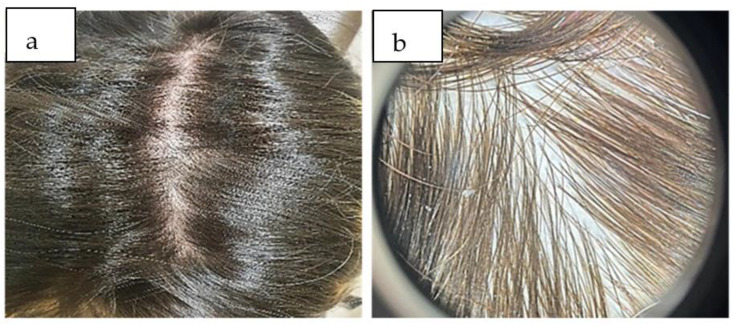
(**a**) A significant amount of hair regrowth after eight months. (**b**) Trichoscopically, the density of hair was wholly recovered, without thin hair.

Eight months after the diagnosis of telogen effluvium, the patient presented with complete recovery of the hair density, as was confirmed trichoscopically (Figure 3a,b).

## 3. Discussion

A meta-analysis carried out in 2022 by Hussain et al. highlighted the most frequent manifestations during the recovery period after COVID-19 to include the following: severe fatigue (58%), headache (44%), impairment of attention (27%), alopecia (25%), and breathing difficulties (24%) [19].

Telogen effluvium is a frequent complication of the COVID-19 infection, which was also described during the 1918 flu pandemic. During the Spanish flu, 2–6 weeks after the start of the disease, diffuse hair loss began. We can note that the onset of alopecia in the context of the flu and the COVID-19 pandemic occurs earlier than in the acute form of telogen effluvium, which appears three months after a trigger factor [19,34].

A retrospective study by Abrantes et al. (2021) on 30 positive COVID-19 patients with TE showed that acute post-COVID-19 telogen effluvium started, on average, 45 days after the infection with SARS-CoV-2. The average duration of the telogen effluvium episode was 47.5 days compared to classic TE, in which the recovery period is 3–6 months [35]. Also, a multicenter study on 214 SARS-CoV-2 positive patients (Moreno-Arrones et al.) showed that TE appeared 57.1 days from the onset of the infection with COVID-19 [36]. Hussain et al. found an average time of 74 days until TE appeared in patients with COVID-19 [19].

Although reports from dermatology clinics decreased during the COVID-19 pandemic, studies and clinical experience have found a higher incidence of telogen effluvium in the post-COVID-19 pandemic period [1,37]. Aksoy et al. showed an upward curve in the incidence of telogen effluvium from 0.5% to 2.3% 3–4 months after COVID-19 became a pandemic infection [38].

The prevalence of telogen effluvium and other hair conditions post-COVID-19 in a series of studies was 20.4% [39]. A meta-analysis performed in Saudi Arabia showed that 48.5% of those studied experienced hair loss of more than 120 hairs/day after infection with COVID-19 [2].

The SARS-CoV-2 virus is involved in triggering telogen effluvium post-COVID-19 both through direct and indirect mechanisms, with systemic and perifollicular inflammatory events. The main mechanisms involved are the following [2]:

- Direct viral damage to endothelial cells: In addition, the SARS-CoV-2 virus can aggravate pre-existing hair conditions by acting on the transmembrane serine protease 2 gene (TMPRSS2)—which has a role in the regulation of androgen pathways [34,40];
- The severe systemic inflammation triggered by the virus influences the appearance of this condition. Because matrix cells are destroyed during the immune response, the cytokine storm can trigger telogen effluvium, and its presence correlates with a higher risk of TE;
- Perifollicular inflammation manifested by the accumulation of activated macrophages and mast cell degranulation in the context of psychological stress [1]. The role of follicular monocytes in triggering the mechanisms of initiation of telogen effluvium is recognized, which determines a transient monocytopenia during the recovery period [41];
- The activation of viral coagulopathy and the formation of microthrombi in the local circulation leads to ischemia and necrosis of the follicles [2] by decreasing the local blood supply [34].

In this inflammatory context, the increase in the circulating level of catagenic cytokines promotes the premature and sudden entry of hair follicles into the catagen phase [1]. Interleukin-6 is the cytokine involved in severe forms of viral infection and telogen effluvium. It predisposes and exacerbates hair loss by inhibiting the first phase of the hair growth cycle (anagen phase) and hair follicle proliferation [39]. In addition, metalloproteinases 1 and 3, together with interleukin-1β, stop hair growth [19].

There is a strong relationship between stress and the onset of this dermatological pathology. Stress inhibits the anagen phase by stimulating the premature entry into catagen and the intrafollicular apoptosis of already-established follicles. The stress and psychological reactions induced by the pandemic facilitated the release of neurotransmitters, neuropeptides, and hormones, thereby negatively affecting the hair cycle [34].

The increased value of cortisol and catecholamines alters the physiological hair cycle, thereby affecting follicular stem cells at the follicular bulb level and dysregulating proteoglycan metabolism at the follicular level [34]. Research has shown that specifically blocking nuclear factor κB (the most important regulator and modulator of inflammation) results in neutralized stress on hair follicles [1]. Blocking the effects of stress hormones and pro-inflammatory cytokines, which influence the activity of follicular cells, can prevent the premature entry of the catagen phase [19].

Some medications used to treat viral infection could trigger telogen effluvium (enoxaparin, hydroxychloroquine, azithromycin, etc.) [42]. On the other hand, there is currently conflicting evidence regarding their mechanisms [19]. Regarding the prophylactic anticoagulant therapy used in patients affected by SARS-CoV-2, most studies reported important associations of hair loss in these patients, but in reality, the World Health Organization (WHO) database provided a smaller number of hair loss cases after warfarin, acenocoumarol, and phenindione use. In these patients, the histopathological examination of the affected areas identified local vascular disorders with the focal degeneration of the collagen fibers at the level of the follicular sheath [43].

In the studies carried out in the population of the Middle East, an important increase in the prevalence of telogen effluvium (in 2/3 of those infected with SARS-CoV-2), of alopecia areata, and of alopecia universalis was found after the anti-SARS-CoV-2 vaccination. This was attributed to the autoimmune reactions triggered by the component substances of the vaccines. However, a direct involvement of these vaccines on the hair growth cycle is not yet known, but it is proven that, in patients vaccinated against SARS-CoV-2, telogen effluvium is the result of the association of chronic viral infection, psychological stress, and certain vaccine components [44].

In our case, establishing the diagnosis of TE was supported by the anamnestic data (COVID-19 infection two months ago), the clinical appearance (the diffuse reduction of capillary density and positive traction tests) corroborated with trichoscopic aspects (decrease in hair density, bare hair follicles, and hairs in the telogen phase), which are aspects similar to those reported in the literature [19].

The most important characteristics of post-COVID-19 telogen effluvium found in the literature, which were consistent in our case as well, are the following:

- A higher incidence of telogen effluvium among women. The studies conducted by Seyfi et al. on 465 patients COVID-19 who tested positive for telogen effluvium showed that 67.5% were women, and the average age was 44 years [34]. In a group of 30 patients, Abrantes et al. highlighted a preponderance of this pathology in women (70%) [35]. Similar data were obtained by Hussein et al. (67.5% women) [19]. Considering the predominance of the condition in women, we can consider that estrogens and progesterone may be involved in the pathogenesis of telogen effluvium. Their effects are immunomodulatory and anti-inflammatory, thus protecting the hair follicle. Estradiol alters the hair follicle growth and cycle through its receptors. Progesterone decreases the conversion of testosterone to dihydrotestosterone, with effects on the hair cycle, thereby shortening the anagen phase. Thus, hair loss in women infected with COVID-19 may be due to the viral infection causing a significant reduction in systemic estrogen and progesterone levels [39]. In a viral context, SARS-CoV-2 increases the level of pro-inflammatory cytokines (IL6—interleukin 6, TNFα—tumor necrosis factor α, and IL1β—interleukin 1β), decreases various growth factors (IGF1—insulin-like growth factor 1, TGF β1—transforming growth factor β1, VEGF—vascular endothelial growth factor, and FGF β—fibroblastic growth factor β), with the result being the apoptosis of follicular keratinocytes.

Moreover, the procoagulant status induced by SARS-CoV-2 causes microthrombi that limit hair’s blood supply, thereby leading to premature entry into the catagen phase [22]. Moreover, in men with androgenetic alopecia, there is a risk of more severe forms of COVID-19, as explained by a vulnerability mediated by genetic polymorphisms of the androgen receptor.

The androgen receptor is the single promoter of transmembrane serine protease 2 (TMPRSS2), which is an enzyme involved in SARS-CoV-2 virus spike protein activation, viral replication, and cell–virus fusion [45]. Other factors include the following:

- The onset of the condition two months after infection: Our patient presented excessive hair loss two months after the SARS-CoV-2 infection. Studies show that 62.5% of patients developed TE within the first month of being diagnosed with COVID-19. and 47.8% developed TE after 12 weeks or more [34].
- Alopecia patterns are diffuse and non-scarring. In our case, the alopecia was diffuse and more evident in the frontoparietal region, in accordance with data from the literature.
- The psycho-emotional implications are significant, especially in female patients. This also motivated our patient to search for a specialized medical service. In addition, the therapeutic response was increased by correcting the emotional component of the patient by correctly informing her about the self-limited and reversible character of this pathology.
- The evolution of the condition is self-limited, with full recovery within a few months of onset. Hussain et al. observed that telogen effluvium is self-limiting, with remission in 3–6 months from the beginning of the onset [19]. However, Seyfi et al. highlighted that excessive, continuous hair loss might occur for over six months [34]. Most patients are concerned that they will gradually lose all the hair on their scalp, and it is important to note that this is not expected, as it has been proven that loss affects up to 30% of scalp hair [46]. The duration and severity of the COVID-19 episode, as well as the severity and the type of hair loss, must also be taken into account, as these are associated with a greater severity of the COVID-19 infection [39]. Considering the importance of the psycho-emotional factor in the etiopathogenesis of this condition, it is of real benefit to inform patients that hair loss is temporary, but, in some cases, it can take up to 18 months for the thickness and density of the hair to return to its original appearance [19].

Regarding the severity of the infectious episode, the data show that most patients with this dermatological manifestation had a severe form of infection requiring hospitalization, systemic therapies (antibiotics, antimalarials), oxygen therapy, or even mechanical ventilation. In moderate and severe forms of infection, pro-inflammatory cytokines are increased, which explains the appearance or worsening of some skin pathologies. In our case, telogen effluvium appeared in a patient with an average COVID-19 infection who did not require respiratory support measures.

This does not rule out the possibility of telogen effluvium in those with asymptomatic COVID-19 infection. Thus, it is mandatory to consider a history of COVID-19 infection, regardless of the severity, to make a differential diagnosis in each patient with telogen effluvium [19].

Abdulwahab et al. consider that the most important factors associated with the appearance of telogen effluvium are female gender, prolonged hospitalization, and a large number of comorbidities: essential arterial hypertension (5.8%), diabetes (4.4%), and respiratory diseases (1.8%) [2]. In our case, the patient had essential hypertension that was under treatment with converting enzyme inhibitors. Consistent with the relationship between hypertension and the severity of COVID-19 infection, the hospitalization rate in those with hypertension was significantly higher than in those without hypertension [38].

In specialized studies, smoking was frequently associated with the appearance of telogen effluvium, especially in those with severe forms of SARS-CoV-2 infection, regardless of gender. Smoking has unfavorable effects on the evolution of the dermatological condition as well as the disease of COVID-19. Nicotine changes the sensitivity of the acetylcholine receptor, thus affecting the hair cycle by influencing the mechanisms of follicular apoptosis [47].

In a study performed on ten patients with TE in the post-COVID-19 period, nine of them had other comorbidities, thus increasing the risk of severe infection with COVID-19, but additional studies are needed to confirm this link [19].

The therapeutic measures for patients with TE post-COVID-19 are not standardized (Table 1). The main therapeutic goal of telogen effluvium is to correct the underlying cause and eliminate the stress factor. Educating the patient regarding the self-limited course of the condition is an essential component of managing post-COVID-19 telogen effluvium [48].

A balanced diet (without refined carbohydrates, sugar and which includes low-fats, vegetables, fruits, healthy fats, and whole grain foods) is a good strategy that can reduce the consequences of hair loss associated with COVID-19 by nutritionally modulating the immune system. Also, the ageusia and/or anosmia encountered in some COVID-19 patients can influence their nutritional status, with deficiencies in essential micronutrients for hair health [46].

The therapeutic measures in post-COVID-19 telogen effluvium are intended to restore the density and thickness of the hairs. The most-used systemic therapies were multivitamins containing zinc, B vitamin complex, iron, selenium, vitamin D3, and biotin. Zinc promotes the regeneration of follicles destroyed by the changes of chronic viral inflammation. Supplementing treatment with vitamin D in COVID-19 patients is beneficial in cases with a low level of 25-hydroxyvitamin D dosage, because it improves the immune system and influences the proliferation of follicular keratinocytes and their entry into the anagen phase. Vitamin E—a powerful antioxidant, inhibits lipid peroxidation by reducing oxidative stress on the scalp [46].

Regarding topical therapies, Minoxidil 2% for women and 5% for men, topical corticosteroids as infiltrations, the injection of plasma rich in platelets (PRP), and mesotherapy can be used [46]. Considering that the effectiveness of mesotherapy in patients with post-COVID-19 telogen effluvium is not established by the FDA, the decision to perform mesotherapy must be individualized [46]. Minoxidil activates the prostaglandin synthase-1 (with hair growth effects) and improves the hair’s blood circulation by local vasodilation, therefore stimulating the transition of hairs from the telogen to anagen phase and extending the anagen phase [46]. The PRP brings benefits by stimulating angiogenesis, increasing the local synthesis of vascular endothelial growth factor (VEGF), and modulating local inflammatory phenomena, thereby preventing the premature entry of the hair into the catagen phase [22]. Mesenchymal and adipose stem cells have been used as regenerative therapies in telogen effluvium in some studies with good results [22]. In order to obtain the most efficient and sustainable results in the shortest period of time, it is necessary to combine different therapeutic methods of hair regeneration. In the case of our patient, the treatment included multivitamins for three months, revitalizing shampoos, and topical therapy with Minoxidil 2% solution twice per day for six months. At the last follow-up (eight months), the patient had a very good emotional status and featured complete recovery of her capillary density, and she was extremely satisfied.

## 4. Conclusions

Every clinician needs to become familiar with the concept of telogen effluvium in the post-COVID-19 period and that it is a common dermatological manifestation that is completely reversible with appropriate dermatological treatment. We know that telogen effluvium is stressful for most patients, even more so for women. Thus, educating the patients about the transitory nature of this condition lessens their stress, blocks their negative feedback related to the pronounced and rapid hair loss, and essentially contributes to the therapeutic success for post-COVID-19 telogen effluvium.

## Figures and Tables

**Table 1:** Treatment options for patients with TE post-COVID-19.

No.	Authors	Years	Number of Patients	Treatment Options
1.	Ohyama et al. [1]	2022	Meta-analysis	Oral or topical minoxidil Oral supplements with biotin, iron, and vitamin D Iontophoresis with growth factors Microneedles
2.	Hussain et al. [19]	2022	465 (meta-analysis)	Oral supplements with sulfur amino acid/vitamin B6, iron, and vitamin D
3.	Saki et al. [49]	2022	1	Oral supplements with vitamin D3 Intramuscular biotin
4.	Lv et al. [50]	2022	1	Topical minoxidil 5% Topical halcinonide lotion Shampoo with selenium sulfide
5.	Olds et al. [48]	2021	10	Oral supplements with biotin, iron, and vitamin D Topical minoxidil 5% Topical corticosteroids
6.	Rizzeto et al. [51]	2021	3	Oral supplements with sulfur amino acid/vitamin B6 Topical peptide (hair growth factor-like) Topical minoxidil 5%
7.	Rossi et al. [4]	2021	14	Oral supplements with biotin, alpha-lipoic acid, iron, vitamin D3 and B5, and topical corticosteroids
8.	Starace et al. [42]	2021	128	Oral supplements with amino acids and vitamins Topical corticosteroids Topical minoxidil Topical hair growth promoters
9.	Moreno-Arrones et al. [36]	2020	214 (meta-analysis)	Oral or topical minoxidil (2% or 5%) Oral supplements Platelet-rich plasma treatment

## Data Availability

The datasets used and analyzed during the present review are available from the corresponding authors upon reasonable request.
